# Supplementary material for: Targeting B4GALT7 suppresses the proliferation, migration and invasion of hepatocellular carcinoma through the Cdc2/CyclinB1 and miR-338-3p/MMP2 pathway
Source: PeerJ. 2023 Nov 21;11:e16450. doi: 10.7717/peerj.16450 (PMC10668818; doi:10.7717/peerj.16450)
Supplement: Supplemental Information 16 [file peerj-11-16450-s016.doc]

Ladder：The ladder was photographed.

With ladder：The ladder was added to the picture.

Repeat: The western blot result was repeated the second time.
